# Interstitial Mycosis Fungoides: An Unusual Mimic of Interstitial Granuloma Annulare Not to Miss

**DOI:** 10.1155/2022/3506738

**Published:** 2022-09-05

**Authors:** Neha Singh, Kiley K. Fagan, Douglas J. Grider

**Affiliations:** ^1^Virginia Tech Carilion School of Medicine, Roanoke, VA, USA; ^2^Section of Dermatology, Department of Internal Medicine, Virginia Tech Carilion School of Medicine, Roanoke, VA, USA; ^3^Department of Basic Science Education, Virginia Tech Carilion School of Medicine, Roanoke, VA, USA

## Abstract

Interstitial mycosis fungoides is a rare histopathologic variant of mycosis fungoides that may resemble interstitial granuloma annulare, inflammatory morphea, and interstitial granulomatous dermatitis. Reported is a case of a 62-year-old African American female who presented with an asymptomatic, progressive rash of the left underarm and abdomen with histologic features suggestive of granuloma annulare. Biopsies revealed an interstitial pattern of cells in the dermis with prominent small aggregates of atypical lymphocytes, a few atypical lymphocytes in the lower epidermis, and a mild increase in dermal mucin. Immunohistochemistry staining revealed the atypical lymphocytes to be positive for CD3 and CD8 and negative for CD4 and CD7, an aberrant immunoprofile. Mixed in the dermis with the atypical lymphoid cells were a few CD68 positive histiocytes and S100 protein positive dermal dendritic cells. T-cell receptor beta gene rearrangement studies showed nearly the same clonal peaks for TCRB rearrangement in two biopsy specimens from separate sites, all supporting a diagnosis of interstitial mycosis fungoides. The patient is undergoing treatment with full body narrowband UVB (nbUVB) phototherapy with notable improvement in skin discoloration and resolution of several abdominal lesions. A diagnosis of interstitial mycosis fungoides is challenging to make based on clinical features alone and is often clinically misdiagnosed. Awareness of histopathologic features is critical to make an accurate diagnosis and thus patient management.

## 1. Introduction

Interstitial mycosis fungoides (IMF), first described by Shapiro and Pinto in 1994, is a rare histopathologic variant of mycosis fungoides (MF) that often presents with erythematous patches and/or plaques of the trunk and proximal limbs. IMF shares many clinical and histological features with interstitial granuloma annulare, inflammatory morphea, and interstitial granulomatous dermatitis, which make it challenging to accurately diagnose. There are several key histopathologic features to make a diagnosis of IMF that have been described in the literature and are emphasized in this report.

### 1.1. Case Report

**Figure 1:**
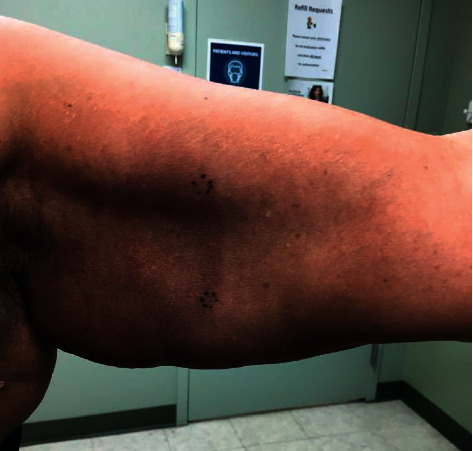
An ill-defined hyperpigmented patch with a wrinkled surface.

**Figure 2:**
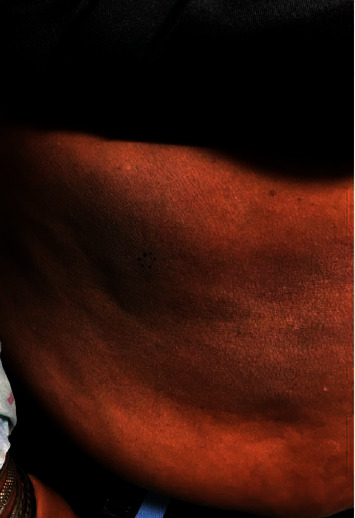
A large, slightly scaling, hyperpigmented patch on the right abdomen.

A 62-year-old African American female presented to the dermatology clinic for a rash present for the past 5 years. It started under her left underarm, and then, she developed various patches on her abdomen that have since grown, covering less than 10% of the body surface area. The rash was asymptomatic but cosmetically bothersome to her. A physical exam revealed a large well-demarcated hyperpigmented thin plaque with a rim of annular erythema extending from the left axilla to the medial upper arm, suggestive of uncertain etiology (Figure 1). Various subtle hyperpigmented ill-defined patches with a wrinkled appearance were present on the lateral right abdomen (Figure 2). The patient had no lymphadenopathy.

**Figure 3:**
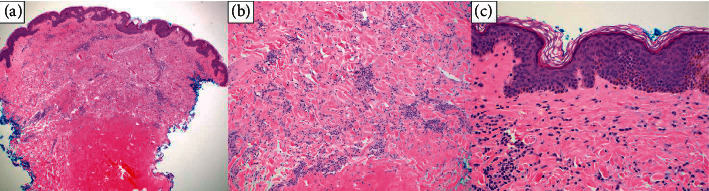
a–c) Lymphocytes, some larger and atypical, in the interstitial pattern in the dermis (hematoxylin & eosin, 4×, 10×, and 20× magnification).

**Figure 4:**
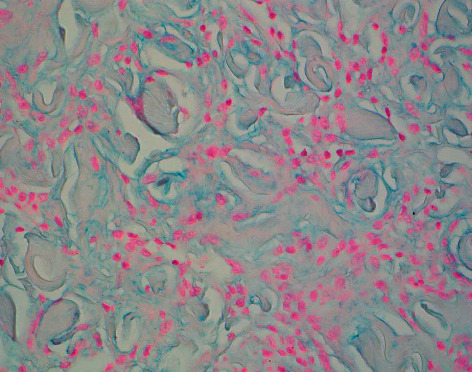
Increased dermal mucin between mononuclear cells (colloidal iron with digestion, 40× magnification).

Three punch biopsies were obtained from the axilla and abdomen, all of which revealed an interstitial pattern of cells in the dermis with prominent small aggregates of mildly enlarged atypical lymphocytes and a few histiocytes associated with a mild increase in dermal mucin (Figures 3(a)–3(c) and 4). There were a few atypical lymphocytes noted in the lower portion of the overlying epidermis. No dermal wiry collagen was present.

**Figure 5:**
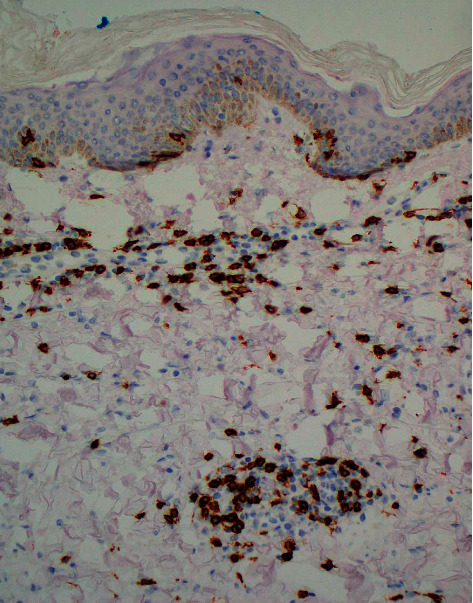
Atypical, enlarged interstitial lymphocytes with positive staining for CD8 in the biopsy from the L axilla (CD8 immunostain, 20× magnification).

**Figure 6:**
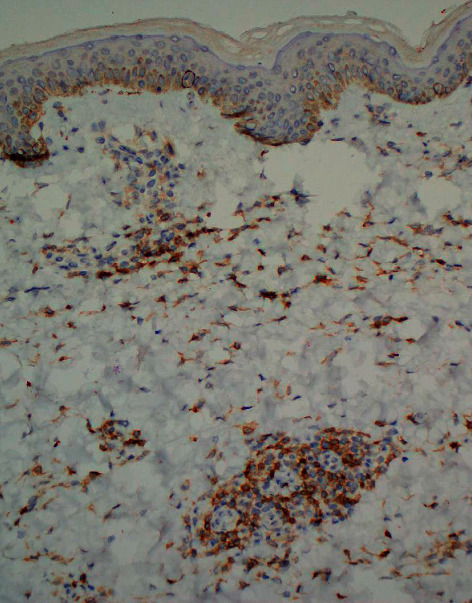
CD4 staining of lymphocytes is decreased in number in the biopsy from the L axilla when compared to CD8 (CD4 immunostain, 20× magnification).

**Figure 7:**
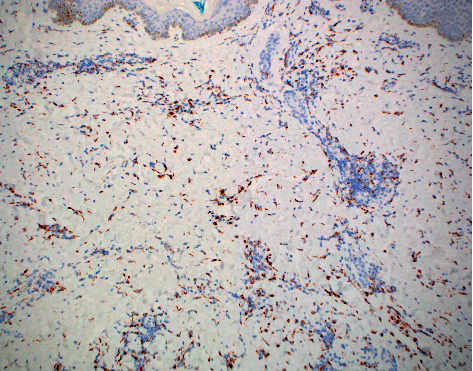
CD68 staining of dermal histiocytes (CD68 immunostain, 10× magnification).

**Figure 8:**
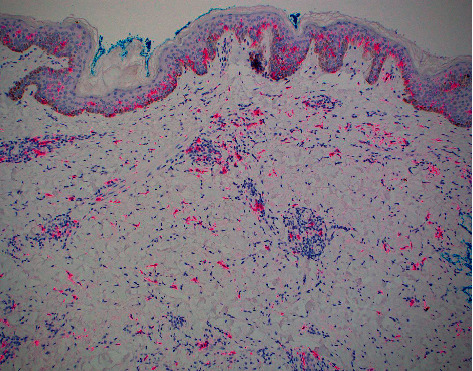
S100 protein staining dermal dendritic cells and Langerhans cells in the epidermis (S100 protein immunostain, 10× magnification).

Ancillary immunohistochemistry demonstrated that the atypical lymphocytes in the dermis stained positive for CD3 and CD8 (Figure 5) and negative for CD4 and CD7 (Figure 6), an aberrant immunoprofile. There were insignificant numbers of CD20 positive B lymphocytes. CD68 stained a few intermixed dermal histiocytes (Figure 7), and S100 protein stained dermal dendritic cells as well as marked Langerhans cells in the overlying epidermis (Figure 8), producing a pattern mimicking interstitial granuloma annulare. T-cell receptor beta (TCRB) gene rearrangement studies showed nearly the same clonal peaks for TCRB rearrangement in biopsies from the left axilla (270, 305) and right abdomen (277, 305).

The patient is currently undergoing treatment with full body narrowband UVB (nbUVB) phototherapy with sessions 2–3 days per week. After 37 sessions, she has shown an improvement in the discoloration of the skin lesion in the left axilla and almost complete resolution of abdominal lesions. She has also been applying hydrocortisone 2.5% cream once daily to these areas.

## 2. Discussion

IMF is a rare histopathologic variant of MF that may resemble interstitial granuloma annulare (IGA), inflammatory morphea, and interstitial granulomatous dermatitis (IGD), and interstitial granulomatous drug reaction [1–7]. Histopathologically, this patient's lesions raised suspicion for granuloma annulare.

The concept of IMF was initially described by Shapiro and Pinto in 1994 and was later reintroduced by Ackerman in 1997 [8, 9]. Shapiro and Pinto reported on the presence of interstitial dermal infiltrates as one of the rare histopathologic patterns of MF and addressed their resemblance to granuloma annulare, while few case series and reports of IMF have been published to date, and they have been observed in both early and advanced stages [1, 9–12]. In the largest published case series to date by Reggiani et al, IMF was found to be a true histopathologic variant of MF with an otherwise conventional clinical presentation [2]. Affected individuals usually display erythematous patches and/or plaques, but macules and nodules have also been described [1, 2]. The disease typically affects the trunk and proximal limbs [1, 2].

Some data suggest IMF may be a transient histopathologic variant of otherwise conventional MF, in which interstitial patterns do not represent the only manifestation of the disease seen in different biopsies taken over time [2, 11]. An interstitial pattern representing the sole histopathologic presentation has been reported in only 2 cases [2]. Histology of IMF reveals prominent, linear aggregates of dermal interstitial infiltrates of small-to-medium sized lymphocytes intermixed with a few histiocytes [2, 12]. Lesion biopsies also show collections of lymphocytes splaying collagen bundles, involving mainly the dermis [1, 2]. In some cases, dermal mucin deposition or follicular mucinosis can be seen [1, 13]. A coarse pattern of fibrosis to the papillary dermis with a lichenoid infiltrate can also be seen in IMF [1].

IMF can also share many similar histopathologic features with MF, including epidermotropism, band-like papillary dermal infiltrates, and clonal T-cell proliferation [2]. Immunohistochemical staining reveals predominantly CD3 positive T cells in the epidermis and dermis with intermixed CD68 positive histiocytes and S100 protein dermal dendritic cells. In a series of 21 patients with IMF, a CD8 positive phenotype was found in 9 of 18 tested cases [2].

The histopathologic patterns detailed above aid in differentiating IMF from other differential diagnoses. In contrast to interstitial granuloma annulare and interstitial granulomatous dermatitis in which CD68 positive histiocytes predominate, IMF usually presents with a predominance of CD3 positive atypical lymphocytes. In addition, these lymphocytes localize to the superficial dermis compared to the deeper dermis seen in interstitial granuloma annulare [1, 8, 10, 12, 14]. IMF can also be distinguished from inflammatory morphea based on the absence of plasma cells, lymphocytes, and histiocytes aggregating along the dermal-subcutaneous junction and absence of sclerosis [15]. Inflammatory morphea also shows superficial and deep, moderately dense interstitial, periadnexal, and perivascular infiltrates of lymphocytes between dermal collagen [1]. Plasma cells are also observed along the dermal-subcutaneous junction.

Based on these nuanced findings, histopathologic evaluation with particular focus on dermal interstitial lymphohistiocytic infiltrates and proper clinicopathologic correlation are necessary for an accurate diagnosis of IMF. This emphasizes the importance of performing a punch biopsy over a superficial shave biopsy, as sufficient depth of the dermis is critical in establishing the diagnosis of IMF [16].

## 3. Conclusion

IMF is a rare histopathologic variant of MF. Clinically and histopathologically, IMF may resemble interstitial granuloma annulare, inflammatory morphea, and interstitial granulomatous dermatitis. Although the clinical behavior, prognosis, and treatment for IMF are similar to classic MF, it is still important for dermatologists and dermatopathologists to be aware of IMF and its mimics because it is easy to misdiagnose.

## Data Availability

The data used to support the findings of this study are available from the corresponding author upon request.
